# LncRNA CCAT1 enhances chemoresistance in hepatocellular carcinoma by targeting QKI-5

**DOI:** 10.1038/s41598-022-11644-4

**Published:** 2022-05-12

**Authors:** Chongsheng Xia, Yurui Sun, Yang Li, Junli Ma, Jing Shi

**Affiliations:** Affiliated Hospital of Jining Medical University, Jining, 272029 Shandong China

**Keywords:** Cancer, Biomarkers, Medical research, Oncology

## Abstract

A major reason for treatment failure of cancer is acquisition of drug resistance. The specific mechanisms underlying hepatocellular carcinoma (HCC) chemoresistance need to be fully elucidated. lncRNAs involve in drug resistance in some cancers, however, the exact functions of lncRNA colon cancer-associated transcript 1 (CCAT1) in oxaliplatin resistance in HCC are still unknown. Our study indicated that CCAT1 promoted HCC proliferation and reduced the apoptosis induced by oxaliplatin. Knockout of CCAT1 could increased chemosensitivity in vitro and in vivo. Further study found that QKI-5 was an important mediator and blocking of QKI-5/p38 MAPK signaling pathway could enhance oxaliplatin sensitivity. In conclusions, CCAT1 promoted proliferation and oxaliplatin resistance via QKI-5/p38 MAPK signaling pathway in HCC. Targeting CCAT1 in combination with chemotherapeutics may be a promising alternative to reverse drug resistance in HCC treatment.

## Introduction

As the fourth most fatal cancer worldwide, hepatocellular carcinoma (HCC) is still a major global health problem^1^. The high mortality is due to late diagnosis with metastasis or poor hepatic reserve. The prognosis of HCC patients, especially those at advanced stages, remains poor. The treatment options, mainly containing targeted therapy and systemic chemotherapy, are limited. Targeted drugs represented by sorafenib, which are clinically approved for the advanced or metastatic HCC, extend survival by very few months^2,3^.

Due to drug costs and availability of targeted drugs, chemotheraputic drugs, especially oxaliplatin, are still adjudged to be effective in a selected population of patients with advanced HCC^4^. However, a major reason for treatment failure is HCC intrinsic or acquired drug resistance. Common mechanisms include drug efflux pumping, enhanced DNA damage repair capacity, inactivation of cell apoptosis and so on^5,6^. And yet, the specific mechanisms underlying oxalipatin resistance need to be fully elucidated.

By influencing gene expression at the transcriptional or post-transcriptional levels, noncoding RNAs (ncRNAs), especially long noncoding RNAs (lncRNAs), were found to be involved in many biological processes, including proliferation, apoptosis, autophagy and metastasis^7,8^. Besides serving as biomarkers of diagnosis in many cancers, lncRNAs involve in drug resistance in multiple types of cancer^9^. Dysregulation of lncRNAs have been reported to be associated with drug resistance and radioresistance. LncRNA HULC was aberrantly expressed in HCC and significantly connected with oxaliplatin, 5-FU and pirarubicin sensitivity through USP22/Sirt1/autophagy pathway^10^. LncARSR was found to promote doxorubicin resistance via modulating PTEN-PI3K/Akt pathway^11^. lncRNA HANR contributed to the development of HCC and enhanced chemosensitivity to doxorubicin by binding to GSKIP^12^.

Colon cancer-associated transcript 1 (CCAT1), located in 8q24.21, is a commonly amplified genomic area in colorectal cancer. Abnormally expressed CCAT1 was associated with aggressive malignancies in multiple types of cancer, including colorectal cancer, breast cancer, squamous cell carcinomas and HCC^13–16^. More importantly, CCAT1 was also regarded as a biomarker for drug resistance in some cancers. It was found that CCAT1 was involved in cisplatin, 5-fluorouracil and paclitaxel resistance^17–19^. However, little is known about the exact functions of CCAT1 in oxaliplatin resistance in HCC. In this study, we identified the possibility that CCAT1 enhanced oxaliplatin resistance and investigated the underlying mechanisms. We found that CCAT1 effectively reduced the chemosensitivity of oxaliplatin in HCC through quaking (QKI)-5/p38 MAPK signaling pathway, providing a new avenue for HCC therapy.

## Methods

### Cell lines and transfection

HCC cell lines HCCLM3 and HepG2 were got from Procell Life Science&Technology Co.,Ltd (Wuhan, China) and cultured in Dulbecco’s modified Eagle’s medium (DMEM, Gibco, Grand Island, NY) containing 10% fetal bovine serum (FBS) in a cell incubator with 5% CO_2_ at 37 °C. pcDNA-CCAT1 plasmid or empty vector was transfected into HCCLM3 and HepG2 cells by Lipofectamine™ 3000 (Invitrogen, Carlsbad, CA) reagent following the manufacturer's protocol. Knocked out CCAT1 in the above cells by CRISPR-CAS9. CRISPR guide RNA specifically targeting CCAT1 sequence (forward gRNA GCCCCTGGCCAACTATATCT; reverse gRNA ATTTGGTCATAATGCGGAAA) was constructed by Genechem (Shanghai, China) and transfected into the above cells.

### qRT-PCR

Total RNA was extracted and reversely transcribed into cDNA using the Reverse Transcription System Kit (TaKaRa Bio Inc., Otsu, Japan). The levels of RNA expression were quantified by qRT-PCR assay (TaKaRa) and calculated by the 2^−ΔΔCt^ method. The values were normalized to an endogenous control GAPDH.

### Chemosensitivity detection

The HCC cells were plated in 96-well plates and exposed to the chemotherapeutic agent of oxaliplatin at the final concentrations of 0, 0.1, 1, 10, 100, 1000 μM for 48 h. Viable cells were determined by cell counting kit-8 (CCK-8, Dojindo, Kumamoto, Japan) according to the manufacturer’s instructions. The cell survival rates and the dose-dependent curves of oxaliplatin were plotted, 50% growth inhibition (IC_50_) values were calculated and analyzed by GraphPad Prism 5.0.

### Apoptosis assay

The assay was conducted by using the Annexin V-FITC Apoptosis Detection Kit (BD Biosciences, San Jose, CA) according to the protocol. Briefly, the treated cells were prepared and incubated with 5 μL Annexin V-FITC and 5 μL PI for 15 min at room temperature in the dark. The cell apoptosis was performed on a Canto II flow cytometry machine (BD Biosciences) within one hour.

### Colony formation assays

The treated HCC cells were seeded into 60 mm dishes with a density of 500 cells/dish and incubated for two weeks. Then the colonies were fixed with ice-cold methanol and stained with 0.1% crystal violet. The numbers of colonies containing more than 50 cells were calculated.

### Immunofluorescence (IF) staining

The treated cells were fixed with 4% paraformaldehyde, permeabilized with 0.1% Triton X-100, blocked with 2% bovine serum albumin and incubated with the primary antibodies against active Caspase3 or QKI-5 at 4 °C overnight. The corresponding Alexa Fluor-conjugated secondary antibodies (Life technology, Waltham, MA) were added, and the nuclei were counterstained by DAPI.

### Detection of caspase-3 and caspase-7 activities

Oxaliplatin-treated cells were plated in 96-well opaque white plate. Caspase 3/7 activities were determined by Caspase-Glo® 3/7 Assay kit (Promega, Madison, WI) according to the manufacturer’s instructions. The blank control, negative control and treated groups were added into 100ul reaction system respectively and cultured for 2 h to being detected the fluorescence value on Promega Glomax.

### Western blot

The protein were separated by sodium dodecyl sulfate polyacrylamide gel electrophoresis (SDS-PAGE) and transferred to PVDF membranes (Millipore, Bedford, MA). After being blocked with 5% skim milk in tris buffered saline (TBS)-Tween 20, the blots were cut prior to hybridisation with primary antibodies, which are listed in Table S1, followed by HRP-conjugated secondary antibodies (Proteintech, Wuhan, China). The immunoblots were visualized by employing the enhanced chemiluminescence kit (Millipore) on a gel imaging analysis system (Tanon, Shanghai, China).

### Cell fractionation assay

HCCLM3 and HepG2 cells were harvested and incubated with a lysis solution. After being centrifuged, the supernatant was used for assessing the cytoplasmic RNA, and the pellet was used for nuclear RNA extraction. GAPDH and U6 were used as cytosolic and nuclear markers respectively.

### In vivo xenograft studies

Twenty BALB/c male mice weighing 18–20 g and aged 4–6 weeks were used (both from Experimental Animal Center of Shandong University, China). All experimental protocols were approved by the Animal Use Committee of Jining Medical Universit. All experiments were performed in accordance with relevant guidelines and regulations. The mice were injected with HCCLM3 and HepG2 cells with CCAT1 knock out and received intraperitoneal injections of oxaliplatin (0.8 mg/kg/w). The tumor growth curves were monitored and the final tumor sizes were calculated using the following formula: V(mm^3^) = (L × W^2^) × 0.5 (L: Tumor length, W: Width).

### Immunohistochemistry (IHC) and a TdT-mediated dUTP nick end-labeling (Tunel) assay

Apoptosis-related proteins (Bcl-2 and Active Caspase-3) were detected in subcutaneous tumor tissues by IHC assay. After deparaffinization, rehydration and antigen retrieval, the sections were incubated with the primary antibodies at 4 °C overnight, followed by the corresponding secondary antibodies. Tunel assay was applied according to the manufacturer's instructions to detect the apoptosis of HCC cells in subcutaneously xenograft tumors.

### RNA pulldown assay

Briefly, cells were lysated and incubated with biotin (Bio)-labeled oligonucleotide probes. The beads were washed and boiled in SDS sample buffer, and the retrieved protein was was validated by standard Western blot analysis.

### Statistical analysis

All statistical analyses were performed with the GraphPad Prism Software (La Jolla, CA, USA). Student’s *t* test was used to analyze differences between two groups, and two-way ANOVA was applied when more than two groups were compared. *p*-values < 0.05 was considered as statistically significant.

## Results

### CCAT1 modulated sensitivity of HCC cells to oxaliplatin

**Figure 1:**
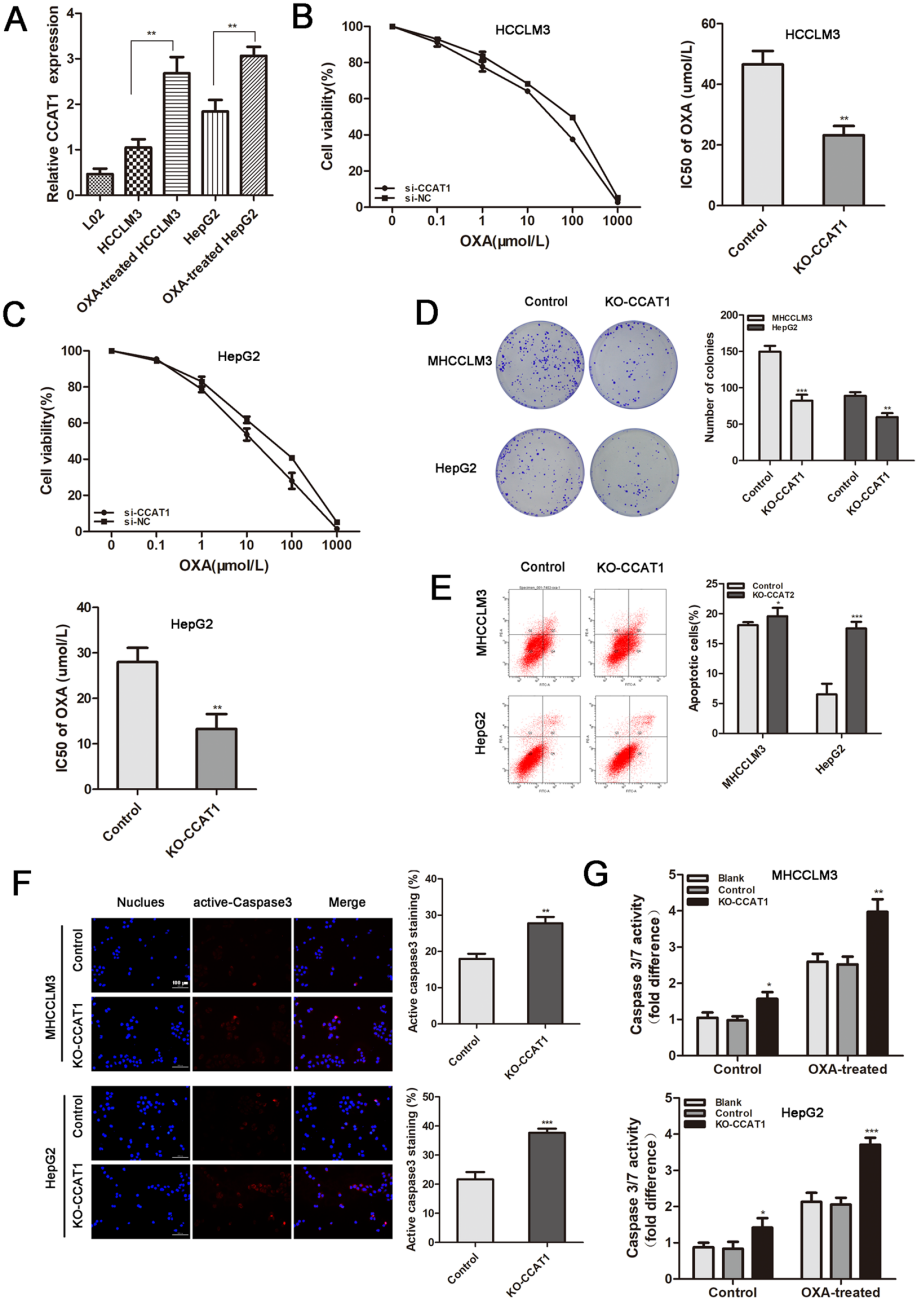
CCAT1 enhanced HCC resistance to oxaliplatin in vitro. (**A**) HCC cells received OXA treatment for 48 h, the expression of CCAT1 was determined by qRT-PCR. (**B**, **C**) HCCLM3 and HepG2 HCC cell lines were knocked out of CCAT1 and exposed to increasing concentrations of oxaliplatin from 0.1 to 1 mM for 48 h to determine the IC50 values by CCK-8 assay. (IC50 of HCCLM3 control and KO-CCAT1: 46.56 *vs.* 23.22 μM; HepG2 control and KO-CCAT1: IC50: 27.98 *vs.* 13.28 μM, *p* < 0.01 respectively). (**D**) The colony formation assay showed that the numbers of colonies were reduced when CCAT1 was knocked out. (**E**) Significantly increased proportion of apoptotic cells by in the KO-CCAT1 groups. (**F**) Significantly increased caspase-3 activation following knock out of CCAT1, indicated by red fluorescence staining (*p* < 0.01). (**G**) Caspase-3 and caspase-7 activities were measured by Caspase-Glo® 3/7 Assay (*p* < 0.05).

We observed that CCAT1 expreesion was significantly higher in oxaliplatin treated cells of HCCLM3 and HepG2 than their parental cells (*p* < 0.01, Fig. 1A). To explore the association between CCAT1 and oxaliplatin sensitivity, we knocked out CCAT1 expressions in HCCLM3 and HepG2 by CRISPR-Cas9. Subsequently, these HCC cell lines with different CCAT1 expression levels were exposed to increased concentrations of oxaliplatin from 0.1 to 1 mM for 48 h to determine the cell viabilities and the IC50 values of oxaliplatin. The results indicated that, compared to the negative control group, knockout of CCAT1 in HCCLM3 showed greater oxaliplatin sensitivity, with lower IC50 values (IC50: 46.56 vs. 23.22 μM, *p* < 0.01, Fig. 1B). As shown in Fig. 1C, HepG2 cells exhibited the same trend (IC50: 27.98 vs. 13.28 μM,* p* < 0.01). The results suggested that there might be a correlation between CCAT1 and oxaliplatin sensitivity in HCC. Then we explored the proliferation and apoptosis of HCCLM3 and HepG2 cells influenced by CCAT1. The colony formation assay demonstrated that CCAT1 knockout could attenuate the numbers of colonies in these two cells, compared to the control groups (*p* < 0.01, respectively. Figure 1D). The flow cytometry showed that knockout of CCAT1 significantly increased the apoptosis of HCC cells, especially in HepG2 treated by oxaliplatin (*p* < 0.001, Fig. 1E). Further, caspase-3 activation in HCCLM3 and HepG2 cells were tested in presence of oxaliplatin at their respective IC50 concentrations. As shown in Fig. 1F, the cells with CCAT1 knockout exhibited higher caspase-3 activation levels (*p* < 0.01). Caspase-Glo® 3/7 assay indicated that caspase-3 and caspase-7 activities were significantly increased when CCAT1 was knocked out, as compared with the control groups both in HCCLM3 and HepG2 cells (*p* < 0.05, Fig. 1G).

### CCAT1 involved in HCC resistance to oxaliplatin in vivo

**Figure 2:**
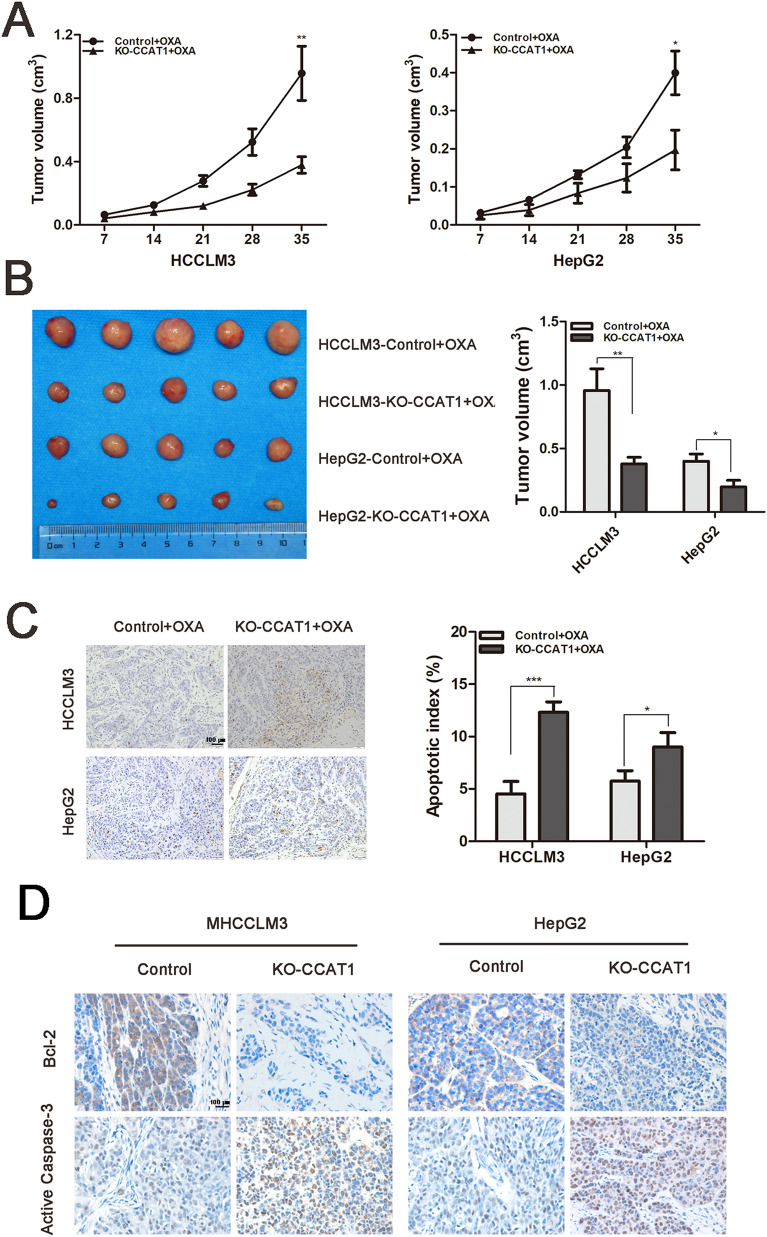
CCAT1 involved in HCC resistance to oxaliplatin in vivo*.* All groups received intraperitoneal injections of OXA (0.8 mg/kg/w). (**A**) Tumor growth curves of subcutaneous implantation models of HCC. (**B**) The average tumor volumes were calculated using the following formula: V(mm^3^) = (L × W^2^) × 0.5 (L: tumor length, W: width). (**C**) TUNEL assay was applied to detect HCC apoptosis in subcutaneous implantation tumor (Original magnification: × 200). (**D**) Immunohistochemistry staining for Bel-2 and active caspase-3 expressions in subcutaneous tumor (Original magnification: × 400). *P < 0.05, **P < 0.01, ***P < 0.001.

To further explore the effects of CCAT1 on HCC chemotherapy in vivo, the stably knocked-out CCAT1 (KO-CCAT1) HCCLM3 and HepG2 cells were used to construct the implantation model. The results indicated that the tumors in CCAT1 knockout groups grew at a significantly slower rate than those in the control groups both in HCCLM3 and HepG2. Five weeks after subcutaneous implantation, the average tumor volume in the KO-CCAT1 groups were significantly smaller than the control groups (0.38 ± 0.05 cm^3^ vs. 0.96 ± 0.17 cm^3^; 0.20 ± 0.05 cm^3^ vs. 0.40 ± 0.06 cm^3^, *p* < 0.05, Fig. 2A,B). Tumor cell apoptosis in the subcutaneous tumors tissue was detected by TUNEL staining. As shown in Fig. 2C, apoptotic cells were significantly increased when CCAT1 was knocked out. The apoptotic rates of HCCLM3 cells in the groups of control and KO-CCAT1 were 4.53 ± 0.67% and 12.30 ± 0.59% (*p* < 0.001) and in the HepG2 cells were 5.73 ± 0.58% and 9.00 ± 0.79% respectively (*p* < 0.05). IHC of tumors tissue was applied to check the expression of apoptosis-related proteins. Higher active caspase-3 and lower Bcl-2 expression levels were observed when CCAT1 was knocked out both in HCCLM3 and HepG2 cells (Fig. 2D).

### CCAT1 interacted with QKI-5

**Figure 3:**
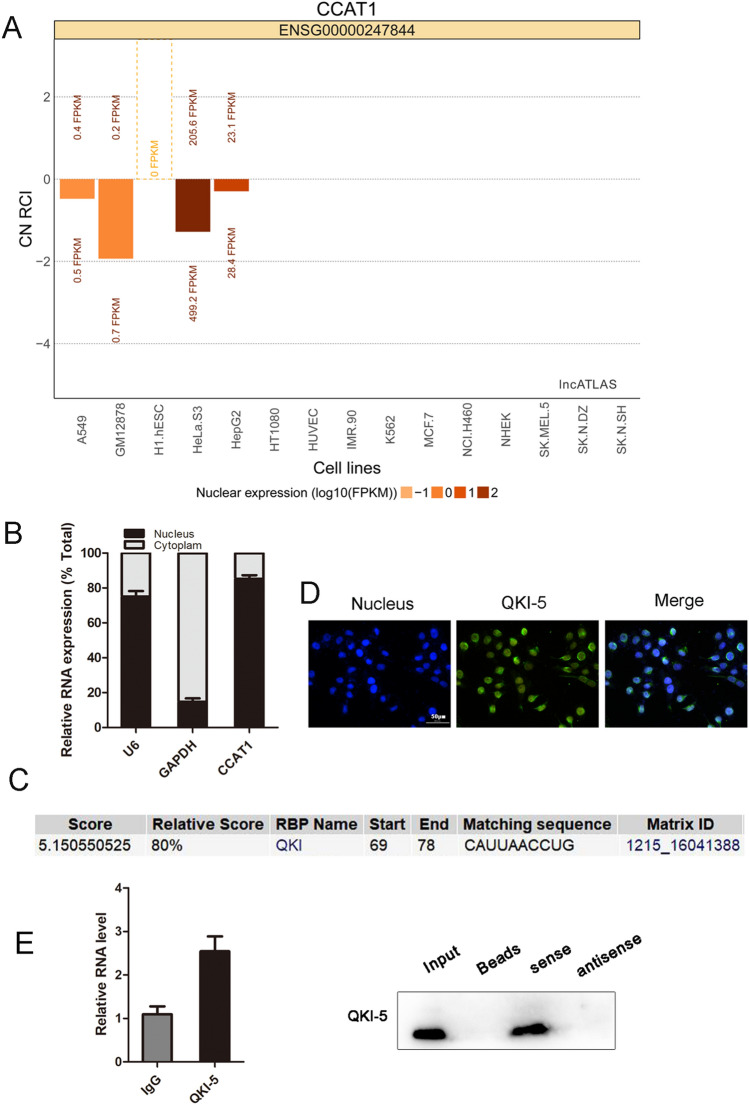
CCAT1 interacted with QKI-5. (**A**) Subcellular localization plots displayed by lncATLAS. (**B**) This nuclear location was confirmed by the cytoplasmic and nuclear extracts measured from qRT-PCR. (**C**) RNA-binding proteins of CCAT1 was predicted by RBPDB (http://rbpdb.ccbr.utoronto.ca/) database. (**D**) Immunohistochemistry staining indicated that QKI-5 was located in the nucleus (Original magnification: × 400). (**E**) CCAT1 was enriched with QKI-5-immunoprecipitation. RNA pull-down assay was applied to detect the QKI-5 antibody.

The subcellular localization of lncRNAs holds valuable clues to their molecular function. CCAT1 is exclusively positioned in the nucleus^20^. Through bioinformatic database of lncATLAS (https://lncatlas.crg.eu/), CCAT1 was observed to be located in nucleus in HepG2 cells (Fig. 3A). We then verified the subcellular localization of CCAT1 by cytoplasmic & cuclear RNA purification (Fig. 3B). LncRNAs could exert their functions through RNA-interacting proteins. Through bioinformatic analysis (http://rbpdb.ccbr.utoronto.ca/), we dentified a set of RNA binding proteins (RBPs) targeted by CCAT1, including QKI (Fig. 3C). As the major isoform of QKIs, QKI-5 was found to be enriched in the nuclei of HCC cells by immunostaining assay, which was consistent with the localization of CCAT1 (Fig. 3D). Further studies were performed to investigate the relationship between CCAT1 and QKI-5, and the results from RIP assay showed the enrichment of CCAT1 in the QKI-5-immunoprecipitation. RNA pull-down assay also confirmed the interaction between CCAT1 and QKI-5 (Fig. 3E).

### Overexpression of QKI-5 reversed oxaliplatin resistance via inhibiting the p38 MAPK signaling pathway

**Figure 4:**
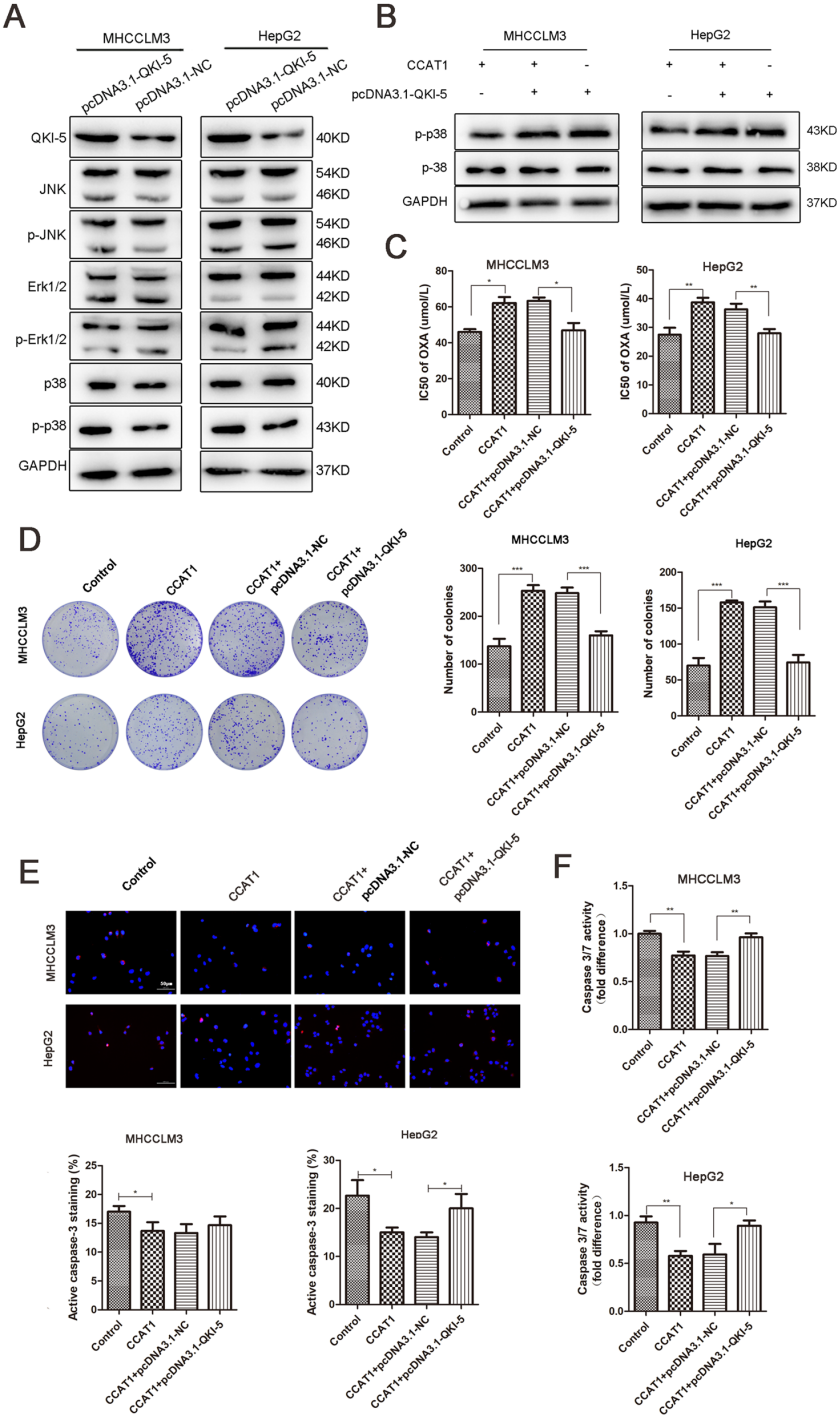
Overexpression of QKI-5 reversed oxaliplatin resistance induced by CCAT1. (**A**) Western blotting analysis of expression levels of QKI-5, JNK, p-JNK, ERK1/2, p-ERK1/2, p38 and p-p38 MAPK in control and pcDNA3.1-QKI-5 cells. (**B**) The phosphorylation and total levels of p38 MAPK in the CCAT1 and QKI-5 overexpression HCC cells. (**C**) IC50 of oxaliplatin was determined when QKI-5 was upregulated. (**D**) The colony formation assay showed that overexpressed QKI-5 significantly attenuated cell proliferation induced by CCAT1 both in HCCLM3 and HepG2 cells. (**E**, **F**) Fluorescence staining and Caspase-Glo® 3/7 Assay showed caspase-3 activity influenced by CCAT1 and QKI-5. Overexpressed QKI-5 could increase caspase-3 activity reduced by CCAT1. *P < 0.05, **P < 0.01, ***P < 0.001.

It is suggested that QKI regulates genes expression through interaction with the quaking response element (QRE). Among the target genes containing at least one QRE, Ras, a member of MAPK signaling pathway, might involve in the regulation^21^. To investigate the major pathway modulated by QKI-5, we upregulated QKI-5 by pcDNA 3.1 plasmid in HCCLM3 and HepG2 cells and detected the expression of JNK, ERK1/2 and p38 by western blotting. The results indicated that the phosphorylation level of p38 MAPK was increased when QKI-5 was overexpressed (Fig. 4A). We found that CCAT1 inhibited the phosphorylation level of p38, while up-regulated QKI-5 could increase p38 phosphorylation level inhibited by CCAT1 (Fig. 4B). Oxaliplatin sensitivity influenced by QKI-5 was explored further. Compared with the control group, CCAT1 dramatically decreased the drug sensitivity, while upregulated QKI-5 could reincrease oxaliplatin sensitivity, with lower IC50 values (IC50 values of HCCLM3: NC: 46.04 ± 1.10 μM, CCAT1: 62.08 ± 2.36 μM, CCAT1 + pcDNA.3.1-NC: 63.31 ± 1.39 μM, CCAT1 + pcDNA3.1-QKI-5: 46.89 ± 2.95 μM; IC50 values of HepG2: NC: 27.49 ± 1.40 μM, CCAT1: 38.768 ± 0.90 μM, CCAT1 + Control: 36.29 ± 1.13 μM, CCAT1 + pcDNA3.1-QKI-5: 27.97 ± 0.85 μM, *p* < 0.01, Fig. 4C). The colony formation assay demonstrated that CCAT1 increased the numbers of colonies, which could be inhibited by up-regulated of QKI-5 (Fig. 4D). This suggests that the simultaneous up-regulated of QKI-5 at least in part revert the effect of CCAT1. Furtherly, cells transfected with pcDNA3.1-QKI-5 exhibited higher caspase-3 activation levels (*p* < 0.01, Fig. 4E,F). These results demonstrate that CCAT1 promoted proliferation and oxaliplatin resistance by QKI-5/p38 MAPK signaling pathway.

## Discussion

Drug resistance is often been activated at the same time when oxaliplatin exerts anti-growth effects. A series of lncRNAs play important roles in chemoresistance and tumor progression. The roles and mechanisms of lncRNAs in drug resistance are complicated. Enhanced drug efflux caused by ATP-binding cassette (ABC) transporters is an important contributor of drug resistance. Up-regulated lncRNA NR2F1-AS1 confers HCC resistance to oxaliplatin by targeting ABCC1^22^. Upregulation of lncRNAs VLDLR and H19 are related with drug sensitivity by inducing the expression of ABCG2 and MDR1^23,24^. The increased ability of DNA damage repair helps cancer cells bypassing the cytotoxicity of chemotherapy drugs. Therefore, the lncRNAs, which involve in genomic stability can modulate drug sensitivity^25^. There are very few researches about CCAT1 and platinum-based chemotherapy. Hu et al.^26^ demonstrated that CCAT1 contributed to DDP resistance by targeting miR-130a-3p/SOX4. Another study suggested that CCAT1 promoted cisplatin-induced apoptosis via CCAT1/miR-454/survivin axis^27^.

120 different lncRNA expression profile in oxaliplatin-resistant HCC cells was identified by microarray^28^. In our study, oxaliplatin resistance induced by CCAT1 in HCC is clarified for the first time. We found that CCAT1 was significantly up-regulated in oxaliplatin-resistant HCC cells. Knockdown of CCAT1 significantly increased the apoptosis of HCC cells and enhanced sensitivity to oxaliplatin and further study demonstrated the role of QKI-5 in this process.

Various factors, including ncRNAs, could regulate QKI. QKIs is a member of the RBPs, including QKI5, QKI6, and QKI7. Through binding with QRE, QKI regulates mRNA stability, RNA transportation and translation. As a major isoform of QKIs, QKI-5 has a nuclear localization signal and is reported to be a tumour suppressor in many cancers^29,30^.

Researches indicate that depletion of QKI‐5 increases cell proliferation and metastasis in oral squamous cell carcinoma, non-small cell lung cancer, renal cell carcinoma and HCC^29,31–33^. What’s more, QKI might involve in drug sensitivity. Some studies showed that overexpression of QKI-5 could attenuate the toxicity of doxorubicin by regulating circular RNAs^34,35^. Yu et al.^36^ demonstrated that 5-FU increased FOXO1 expression via inhibition of QKI in the oncogenesis of breast carcinoma. However, the roles of QKI in oxaliplatin resistance remain to be fully elucidated.

OXA treatment form platinum–DNA adducts, which can’t be repaired by mismatch repair (MMR) system. Nucleotide excision repair (NER) is the main DNA damage repair pathway. In NER, the most important mediums are excision repair cross complementary 1 (ERCC1) and its catalytic companion XPF (ERCC4). Bioinformatic analysis by star Base indicates that QKI and ERCC1 have binding sites, and ERCC1 might be the target gene of QKI. So we speculate that CCAT1/QKI/ERCC1 might play import role in DNA damage and oxaliplatin resistance, which need to be verified in our future work.

## Conclusions

In our investigation, we have established a signaling cascade of CCAT1 and QKI-5 in the regulation of HCC cell proliferation and chemoresistance. Targeting lncRNAs in combination with chemotherapeutics may be a promising alternative to reverse drug resistance in cancer treatment.

## Supplementary Information

Supplementary Information 1.

Supplementary Information 2.

Supplementary Information 3.

Supplementary Information 4.
