# Supplementary material for: LncRNA CCAT1 enhances chemoresistance in hepatocellular carcinoma by targeting QKI-5
Source: Sci Rep. 2022 May 12;12:7826. doi: 10.1038/s41598-022-11644-4 (PMC9098857; doi:10.1038/s41598-022-11644-4)
Supplement: Supplementary file 1 — Supplementary Information 1. [file 41598_2022_11644_MOESM1_ESM.doc]

**Supplementary Table S1：The types, dilutions and sources of antibodies used for the Western blot and immunohistochemical analysis.**

| **Antibody** |  | **Working dilution** |  | **Working dilution** |  | **Species** | **Source -Cat. Number** |
| --- | --- | --- | --- | --- | --- | --- | --- |
|  |  | **Western blot** |  | **IHC** |  |  |  |
| Bcl-2 |  | - |  | 1:500 |  | Rabbit | abcam  (cat. No. ab32124) |
| Active caspase-3 |  | - |  | 1:200 |  | Rabbit | abcam  (cat. No. ab32042) |
| JNK |  | 1:1000 |  | - |  | Rabbit | Cell Signaling Technology  (cat. No. #9926T) |
| p-JNK |  | 1:1000 |  | - |  | Mouse | Cell Signaling Technology  (cat. No. #9255S) |
| Erk1/2 |  | 1:1000 |  | - |  | Rabbit | Cell Signaling Technology  (cat. No. #9926T) |
| p-Erk1/2 |  | 1:1000 |  | - |  | Rabbit | Cell Signaling Technology  (cat. No. #4370S) |
| P38 |  | 1:500 |  | - |  | Rabbit | Cell Signaling Technology  (cat. No. #9926T) |
| p-p38 |  | 1:1000 |  | - |  | Rabbit | Cell Signaling Technology  (cat. No. #4511T) |
| QKI-5 |  | 1:1000 |  | - |  | Rabbit | Millipore Sigma  (cat. No. AB9904) |
| GAPDH |  | 1:2000 |  | - |  | Rabbit | abcam  (cat. No. ab9485) |
