# Supplementary figures and images for: LncRNA CCAT1 enhances chemoresistance in hepatocellular carcinoma by targeting QKI-5
Source: Sci Rep. 2022 May 12;12:7826. doi: 10.1038/s41598-022-11644-4 (PMC9098857; doi:10.1038/s41598-022-11644-4)

QKI-5

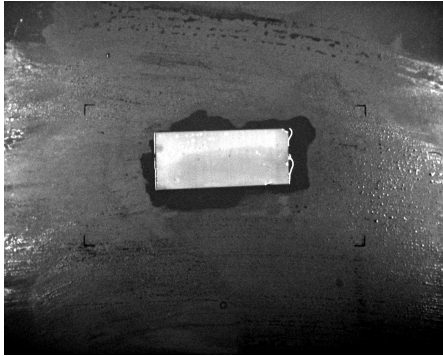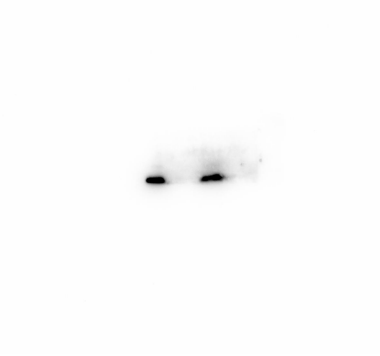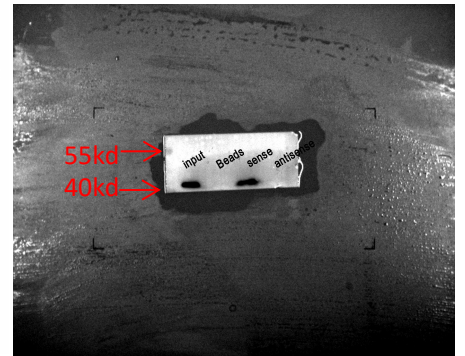

Supplement: Supplementary file 2 — Supplementary Information 2. [file 41598_2022_11644_MOESM2_ESM.pdf]
