# Supplementary material for: LncRNA CCAT1 enhances chemoresistance in hepatocellular carcinoma by targeting QKI-5
Source: Sci Rep. 2022 May 12;12:7826. doi: 10.1038/s41598-022-11644-4 (PMC9098857; doi:10.1038/s41598-022-11644-4)

**Original data of western bands in Figure4A (MAPK pathway)**

The western experiments were performed on the gel imaging system (Tanon, Shanghai, China), so the membranes images (white, brand and merge) were get directly.

The blots were cut prior to hybridisation with antibodies during blotting, which were clarified in the methods.

**Lanes 1-2: HCCLM3;    lanes 3-4:HepG2**

QKI-5 band （40kd）

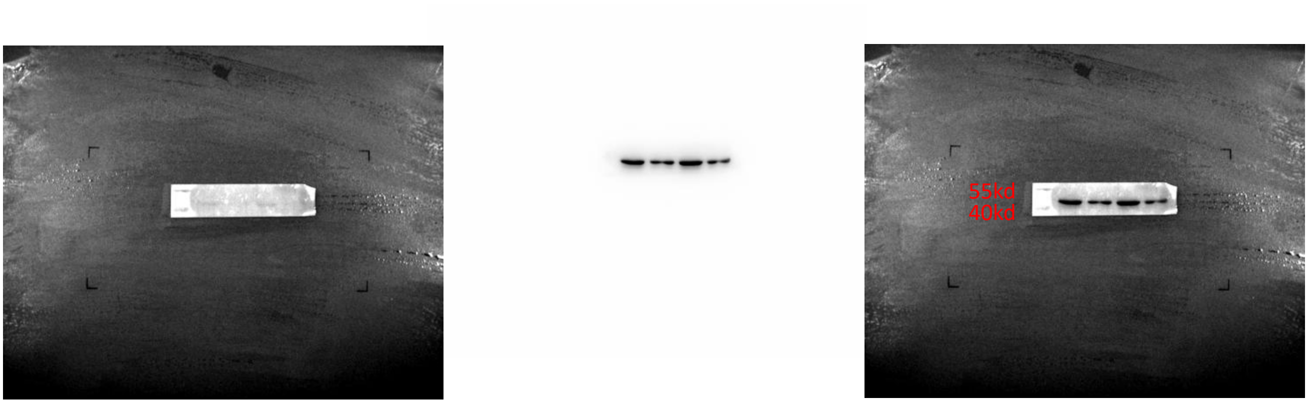


Erk（42/44kd）

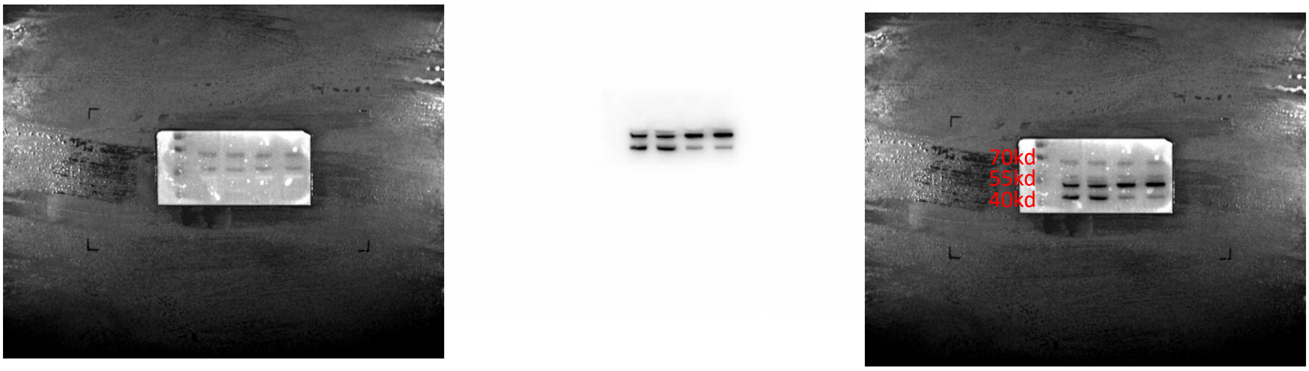


P-Erk（42/44kd）

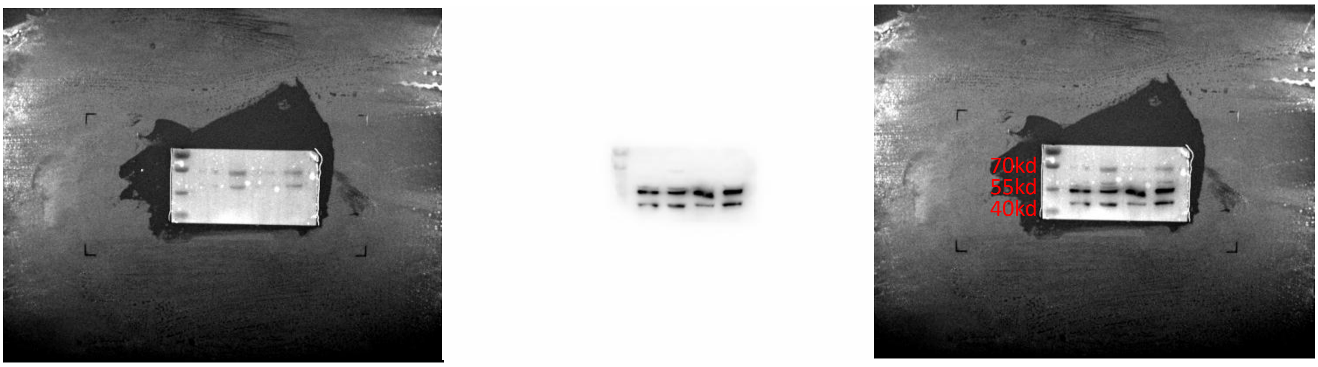

**JNK（46/54kd）**

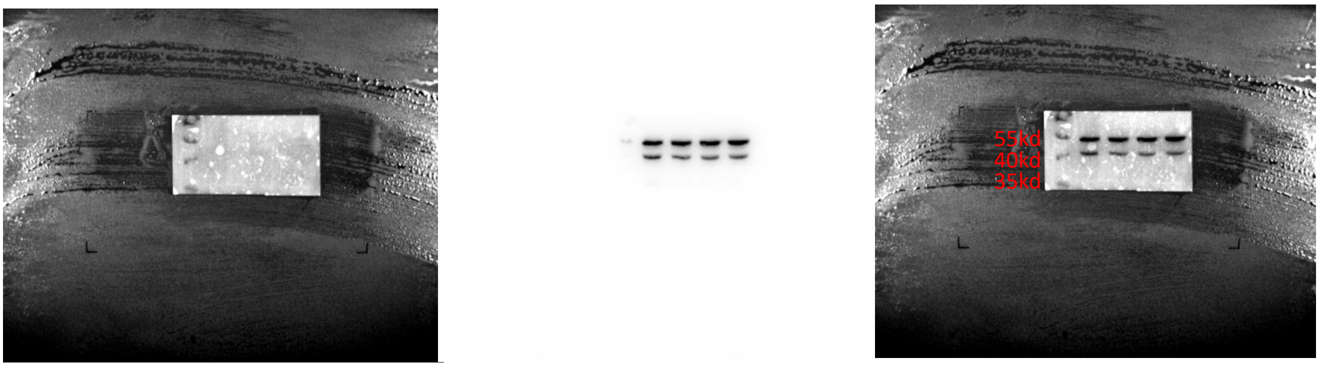


P-JNK（46/54kd）

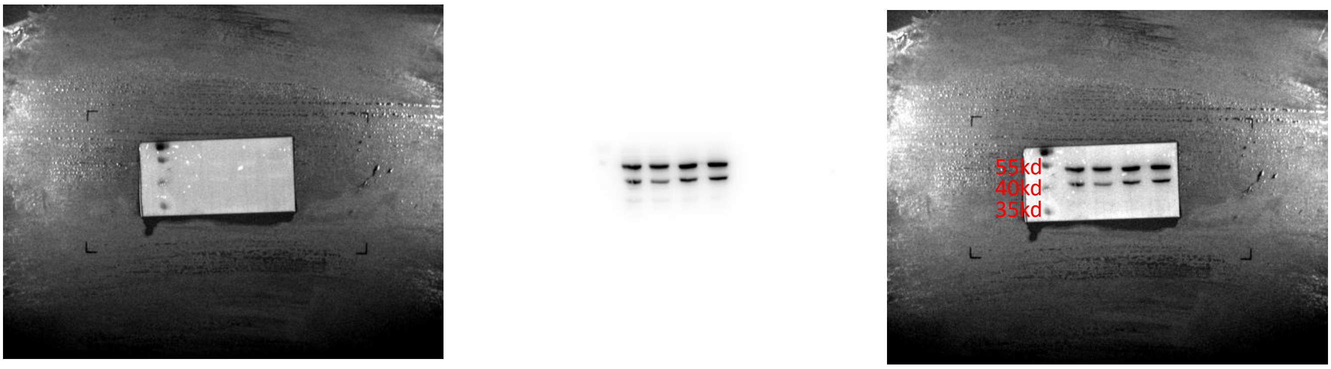


P38（40kd）

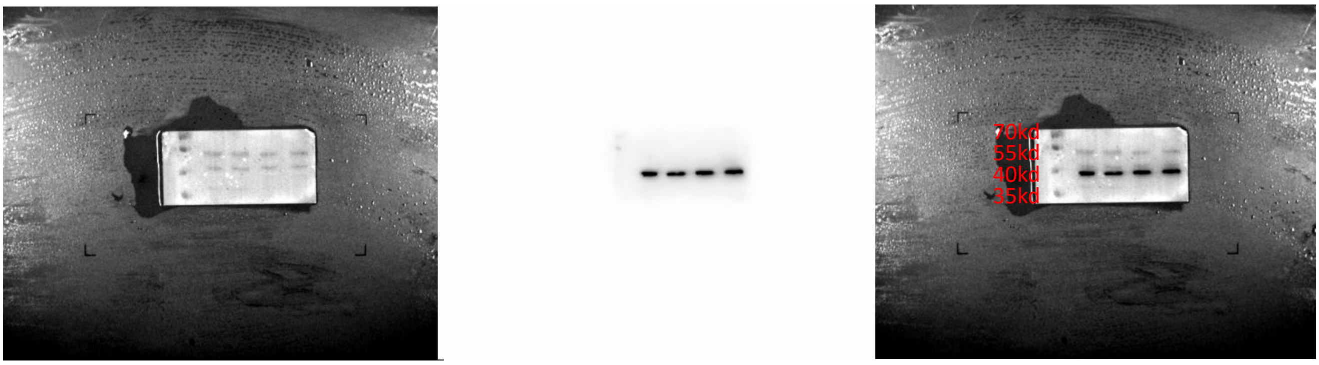


P-p38（43kd）

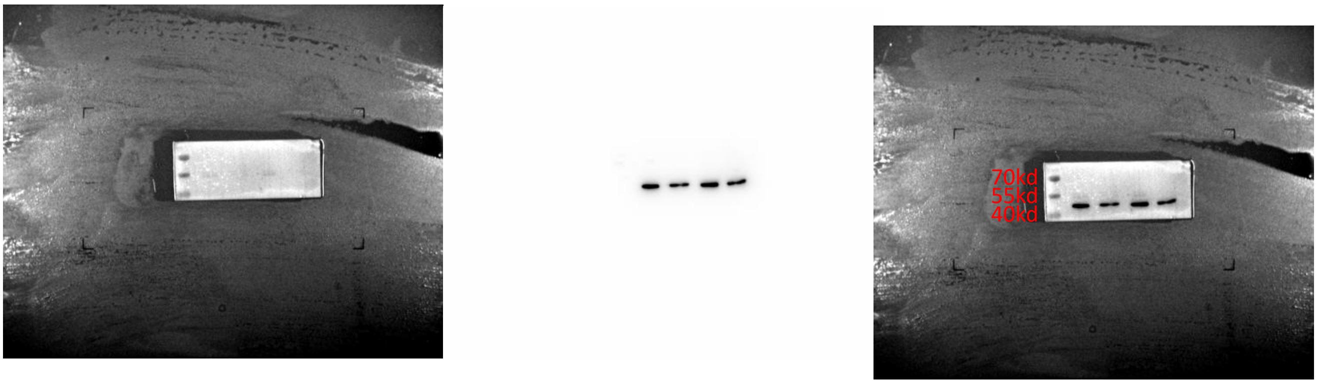

Gapdh

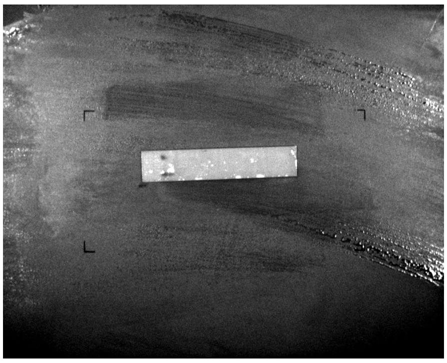

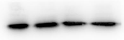

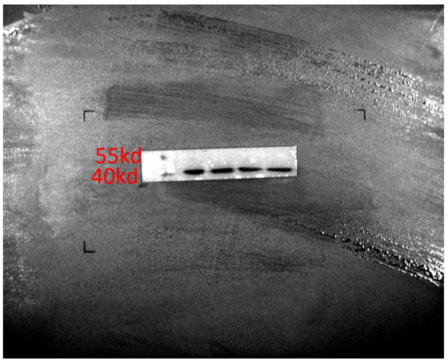

Supplement: Supplementary file 3 — Supplementary Information 3. [file 41598_2022_11644_MOESM3_ESM.pdf]
